# Supplementary material for: State of biodiversity documentation in the Philippines: Metadata gaps, taxonomic biases, and spatial biases in the DNA barcode data of animal and plant taxa in the context of species occurrence data
Source: PeerJ. 2022 Mar 21;10:e13146. doi: 10.7717/peerj.13146 (PMC8944339; doi:10.7717/peerj.13146)
Supplement: Supplemental Information 1 — This file contains the different the supplementary tables for the manuscript and the supplementary figures, which are the same figures presented in the main text but using different data. [file peerj-10-13146-s001.docx]

# **Supplemental Tables**

**Table S1.** Categories of vague issues encountered, and their corresponding definitions and criteria, while manually parsing through entries found in GenBank and Barcode of Life Data System (BOLD) on the descriptive information on sampling locality, focusing particularly on the administrative units of province, municipality, and barangay.

| **CATEGORY** | **CRITERIA** |
| --- | --- |
| **none** | No major issues encountered |
| **unspecified** | All/some information provided does not fall into any of the admin unit categories (i.e., province, municipality, barangay) but still somewhat informative and is either of the ff:  - only indicate a general region, such as seas or particular islands (e.g., Luzon, Mindanao, Panay, Mindoro Island, Negros Island, etc.)  - referring to a location (e.g., geographic features such as river, mountain, mountain range; conservation areas such as national/natural parks; or public markets) that is either vague/common or unique but covers a vast area and so, unable to pinpoint which province (at the very least) is being referred to  - lacking details such as "Mt." or "Lake" to indicate what kind of location (i.e., geographic feature, administrative unit, institution, dive site) the name is referring to  Also refer to cases where name is given and is found in the PH admin database, however, lacks details about the specific administrative unit (such as province and/or municipality) in which the location is found. such as those with multiple municipalities/barangays having the exact same name and no other specific details are provided to pinpoint which exact location being referred to |
| **misspelled** | Includes either of the ff:  - incorrect spelling (e.g., Sebu instead of Cebu)  - the name of place is incomplete based on the names in the PH admin unit (e.g., with the exceptions of alternative names and minor details such as "City of" or "Capital") |
| **unknown** | All/some information provided does not fall into any of the admin unit categories (i.e., province, municipality, barangay) but is unclear and not informative because it is either:  - Difficult to find more about the provided location details (i.e., unable to find more information about the location through search engines such as Google or the journal article associated with the GenBank entry)  - Difficult to understand and unsure if the given information has been shortened, misspelled, or referring to some acronym  - Questionable if information provided is referring to geographic location or may be referring to names of people or other things |
| **mismatch** | Information has a discrepancy between the country, province, municipality, and/or barangay provided |
| **multiple** | More than one entry for a particular admin unit and so, may pertain to two or more different locations |
| **mixed** | More than one issue was observed while parsing through an entry |

**Table S2.** Metadata information pulled out from the following databases: Global Biodiversity Information Facility (GBIF), Barcode of Life Data System (BOLD), and GenBank. Metadata were categorized as taxonomic, publishing, records, sequence, and geolocation information. Those aligned in the same row represent analogs. Some columns, as indicated by an asterisk (*), required manually editing and parsing. Note that for the metadata with “phylum” in their column label, they include taxa that are classified as divisions in plant taxonomy.

| **GBIF** | **BOLD** | **GenBank** |
| --- | --- | --- |
| ***Taxonomic Information*** | | |
| kingdom | kingdomUse* | |
| phylum | phylum_name | phylum* |
| class | class_name | class* |
| order | order_name | order* |
| family | family_name | family* |
| genus | genus_name | genus* |
| species | species_name | organism* |
| ***Publishing Information*** | | |
| publisher | institution_storing | PublishingInstitution* |
|  | copyright_institutions |  |
|  | sequencing_centers |  |
| publishingCountry | storing_country* | PublishingCountry* |
|  | copyright_country* |  |
|  | sequencing_country* |  |
| ***Records Information*** | | |
| eventDate | collectiontime | collection_date* |
| gbifID | genbank_accession | Accession* |
|  | processid | boldInfo* |
| – | – | yearSubmitted* |
| ***Sequence Information*** | | |
| – | nucleotides | Sequence* |
| – | markercode | StdGeneMarker* |
| ***Geolocation Information*** | | |
| decimalLatitude | lat | lat* |
| decimalLongitude | lon | lon* |
| countryCode | country_edited* | country_edited* |
| province_final* | province* | province* |
| municipality_final* | municipality* | municipality* |
| barangay_final* | barangay* | barangay* |
| – | major_issues* | major_issues* |

* = manually parsed and edited

**Table S3.** Characterization of available Philippine species occurrence and DNA barcode data in terms of overall records initially collected and analyzed as well as the associated genetic markers of interest and taxonomic classification (focusing on the higher taxonomic levels). Because two sets of analyses were conducted – including and excluding barcode records with *NA* entries for country sampled two sets of summaries are also presented to characterize the DNA barcode data available.

| **Categories** | **Species Occurrence Data** | | **DNA Barcode Data** | | | |
| --- | --- | --- | --- | --- | --- | --- |
|  |  |  | ***NA include*** | | ***NA exclude*** | |
| Total Records |  | | | | | |
| *Collected* | 1,557,709 | | 31,163 | | | |
| *Analyzed* | 1,557,374 | | 20,482 | | 16,719 | |
| Records per Genetic Marker |  | | | | | |
| *COI* | – | | 14,755 | | 12,988 | |
| *CYTB* | – | | 3,731 | | 2,095 | |
| *ITS2* | – | | 1,059 | | 827 | |
| *matK* | – | | 314 | | 268 | |
| *rbcL* | – | | 623 | | 541 | |
| Records per Kingdom |  | | | | | |
| *Animalia* | 1,229,860 | | 18,705 | | 15,217 | |
| *Plantae* | 327,514 | | 1,775 | | 1,500 | |
| Unique Taxa per Classification | *Animalia* | *Plantae* | *Animalia* | *Plantae* | *Animalia* | *Plantae* |
| *Phylum/Division* | 25 | 8 | 15 | 5 | 15 | 5 |
| *Class* | 74 | 27 | 35 | 13 | 34 | 13 |
| *Order* | 414 | 142 | 163 | 61 | 159 | 58 |
| *Family* | 2,949 | 509 | 566 | 130 | 550 | 115 |

# **Supplemental Figures**


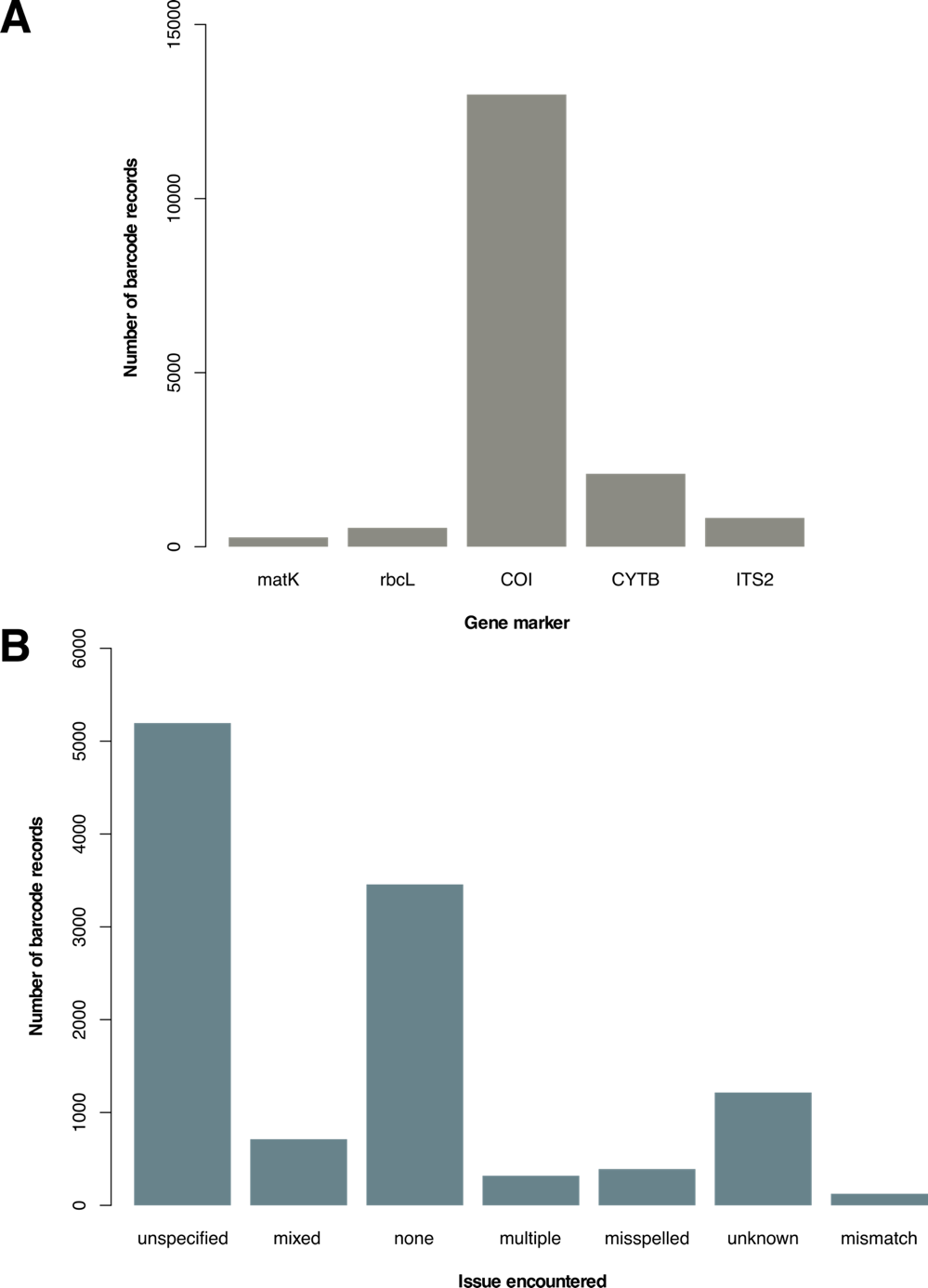


**Figure S1.** **Summary of barcode records associated with specific gene markers and issues encountered while manually parsing through the descriptive information on sampling locality.** For graph **A**, the genetic summary of the available barcode records focuses on the gene markers of interest used in the examination for metadata gaps, taxonomic biases, and spatial biases in DNA barcode data on animal and plant taxa sampled in the Philippines were the following: cytochrome b (CYTB), cytochrome oxidase c subunit I (COI), internal transcribed spacer 2 (ITS2), ribulose-1,5-biphosphate carboxylase (rbcL), and maturase K (matK). For graph **B**, the geolocation issues resulted in the descriptions of the sampling location (particularly in terms of administrative units) being unclear or in some cases, inconclusive. The categories include misspelled (incorrect spelling), none (no major issue), mixed (more than one issue), unspecified (somewhat informative but still vague), unknown (completely not informative), multiple (provided more than one location), and mismatch (discrepancies between the administrative units provided). The dataset excludes the records with *NA* entries for country sampled (for **A** and **B**) and those that had additional information on the geolocation other than the coordinates (for **B** only).


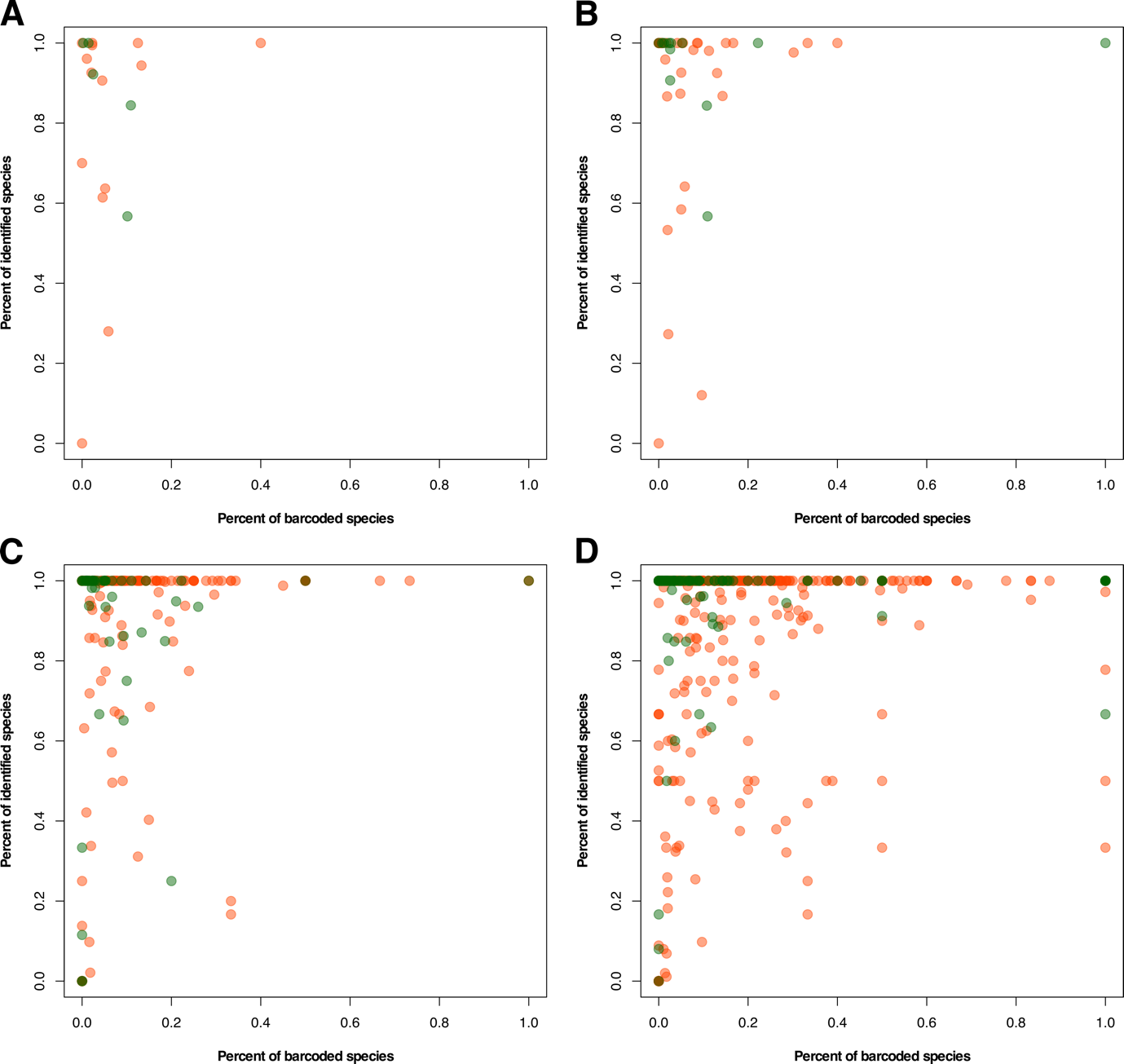


**Figure S2.** **Relationship between the percentage of barcode records identified at the species level and the proportion of documented species (represented in species occurrence data) that currently have DNA barcode data available.** This relationship was evaluated for each known animal (orange) and plant (green) taxonomic group represented in the Philippine barcode data at the phylum/division (A), class (B), order (C), and family (D) levels. This dataset excludes the records with *NA* entries for country sampled.


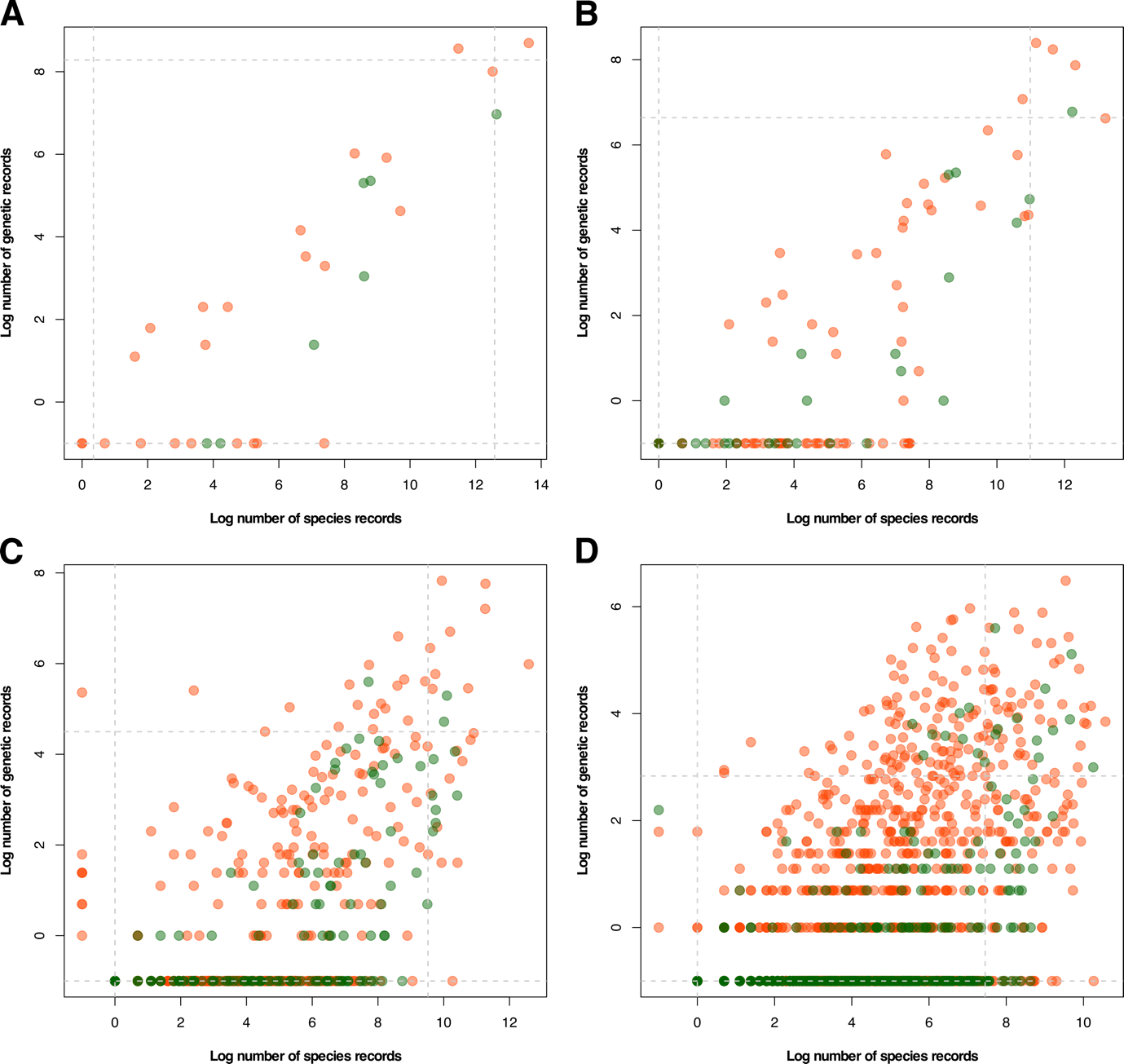


**Figure S3.** **Relationship between the amount of genetic and species data associated with each known animal and plant taxonomic group represented in the Philippine biodiversity data at different taxonomic levels.** This relationship was evaluated for each known animal (orange) and plant (green) taxonomic group represented in the Philippine barcode data at the phylum/division (A), class (B), order (C), and family (D) levels. Values were transformed logarithmically prior to plotting however, taxa with zero (0) records in either genetic or species data were assigned the value of negative one (-1). Dashed lines represent the 5th and 95th percentiles for genetic (horizontal) and species (vertical) data. This dataset excludes the records with *NA* entries for country sampled.


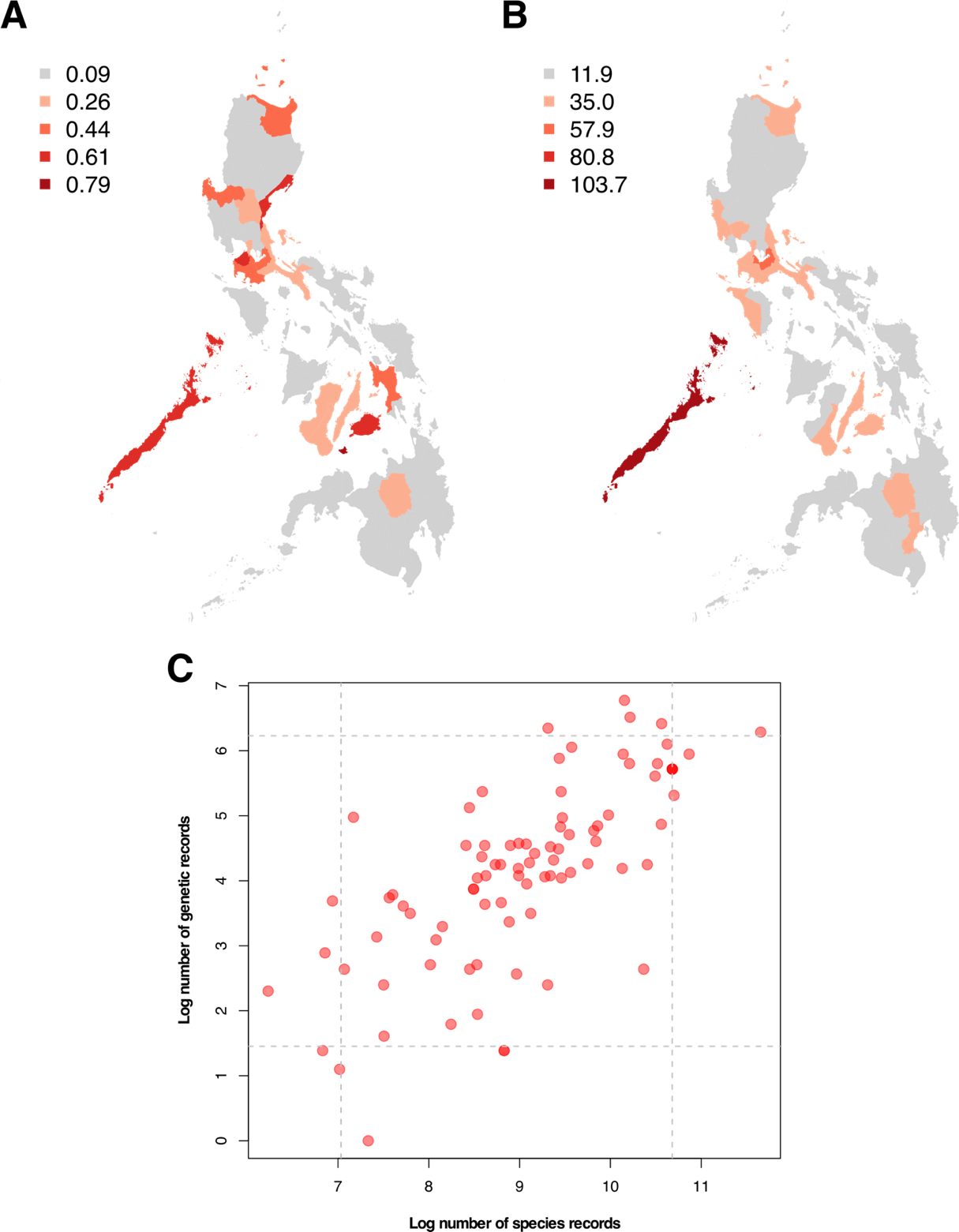


**Figure S4.** **Maps of the sampling distribution of barcode and species occurrence data on animal and plant taxa across the Philippines and the relationship between the two datasets in terms of province.** For both maps (**A** – barcode data and **B** – species occurrence data), records on marine specimens were assigned to a specific province based on which corresponding centroid has the shortest distance from the given sampling coordinates (if available). Also, values presented in the maps represent the number of records in the thousands. In the scatter plot (**C**), values were transformed logarithmically and provinces with zero (0) records in either genetic or species data were assigned the value of negative one (-1). Dashed lines represent the 5th and 95th percentiles for genetic (horizontal) and species (vertical) data. The barcode dataset excludes the records with *NA* entries for country sampled.


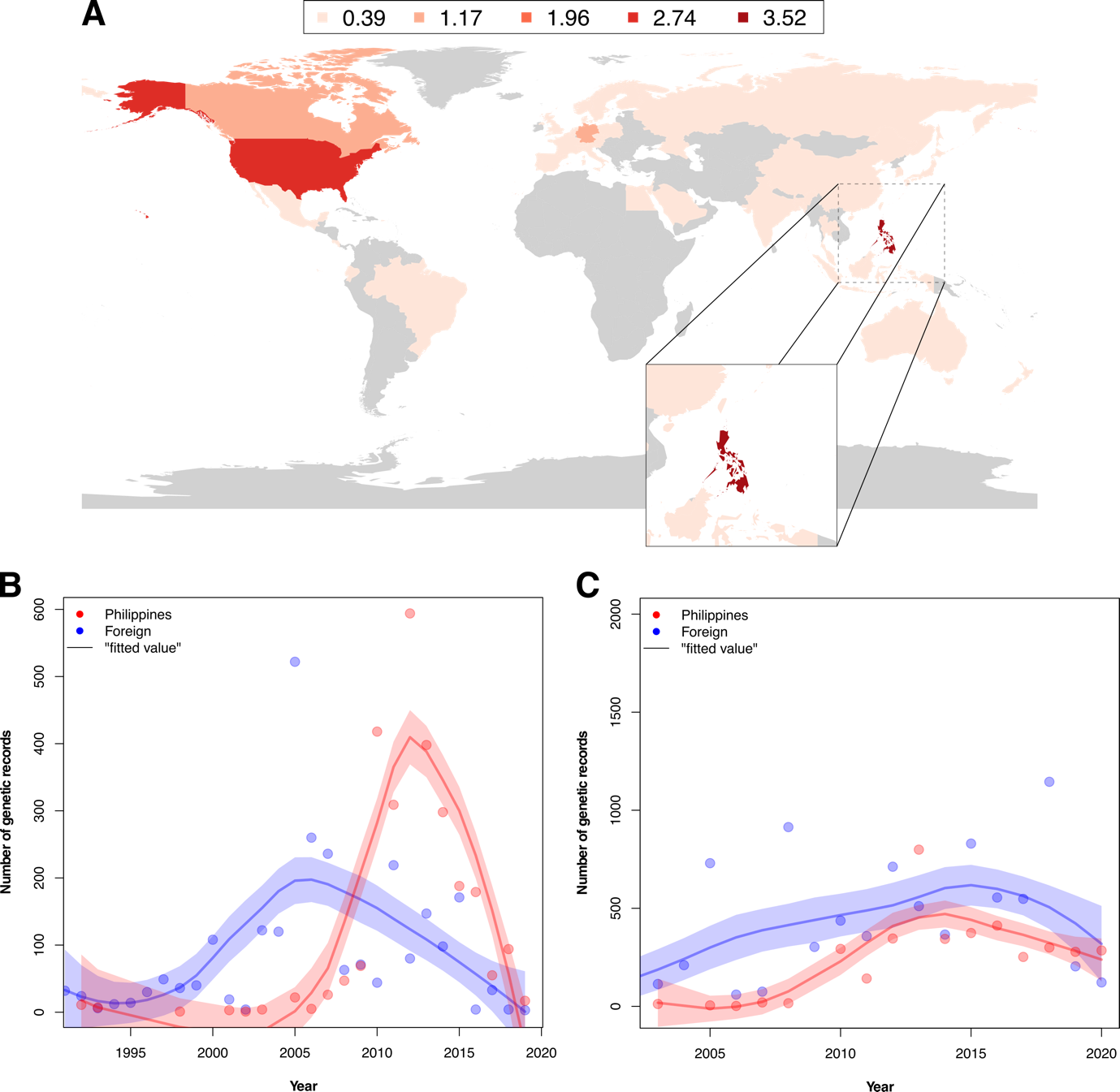


**Figure S5.** **Map of the distribution of barcode data on Philippine animal and plant biodiversity contributed by different countries across the world and their contribution to documenting efforts across the years.** For map **A**, contribution was based on the institution that holds the copyright to the image associated with the records while for the graphs, it was based on the collection of samples, starting from the 1990s (**B**) and submission of barcode data, starting from the 2000s (**C**) by foreign countries (violet) and the Philippines (red). Trendlines in the graphs represent the average, “best” fitted line. This dataset excludes the records with *NA* entries for country sampled.


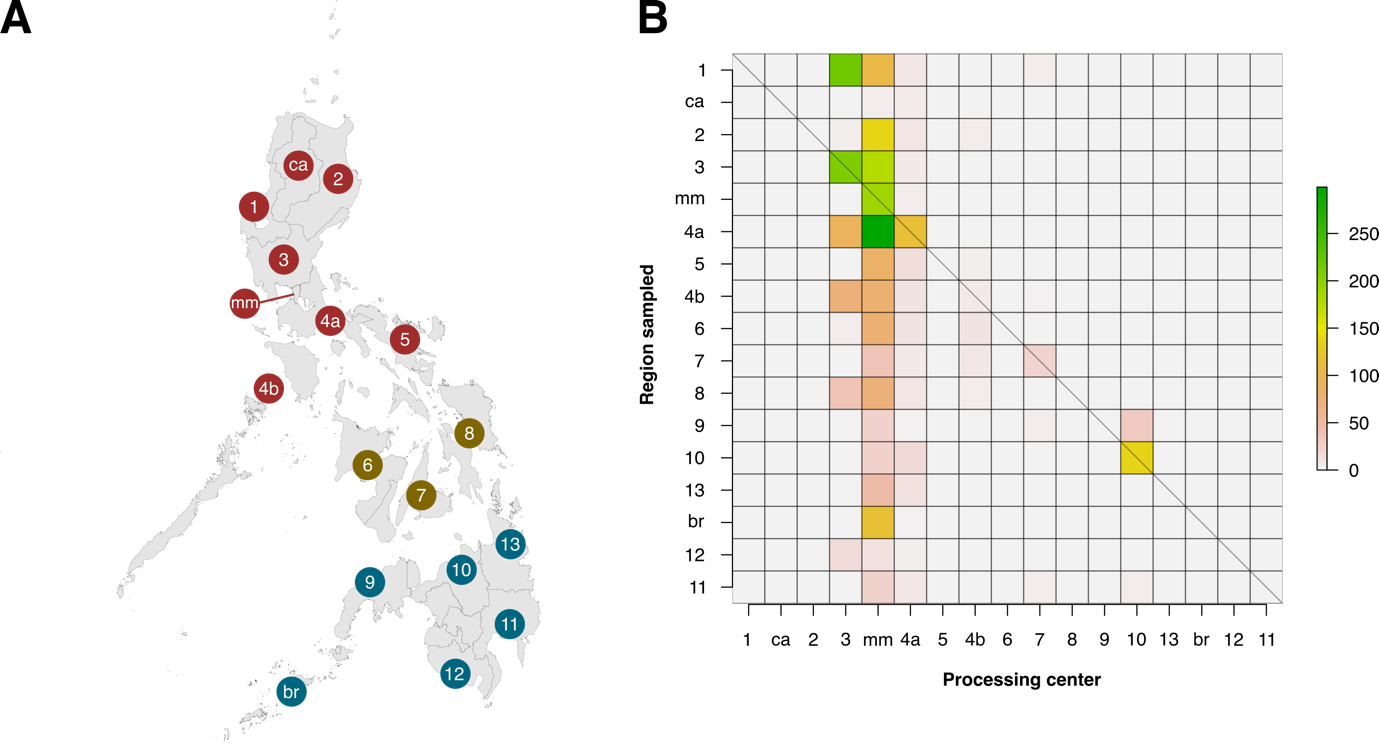


**Figure S6**. **Heatmap matrix showcasing the relationship between the number of barcode records associated with regions that have been sampled and the regions of local institutions that contributed the data.** There are officially seventeen regions in the Philippines, represented by the Philippine map (**A**), with non-numerical regions labelled as follows: *ca*, Cordillera Administrative Region (CAR); *mm*, National Capital Region (NCR or also referred to as Metro Manila); and *br*, Bangsamoro Autonomous Region in Muslim Mindanao (BARMM). Regions are also divided based on their island groups – namely Luzon (red), Visayas (yellow), and Mindanao (blue). For matrix **B**, contribution was based on the institution that holds the copyright to the image associated with the records. Regions along the x- and y-axis are sorted to provide spatial context, with the map as a reference. The diagonal line represents the “ideal” scenario wherein the region serving as the processing center of barcode data can sufficiently sample its own local area. This dataset excludes the records with *NA* entries for country sampled.
